# Surface Engineering of Non-Equiatomic TiZrNbTaMo HEA by MAO Treatment in a Cu-Rich Electrolyte for Biomedical Applications

**DOI:** 10.3390/ma19010174

**Published:** 2026-01-03

**Authors:** Samuel P. Bonetti, Jhuliene E. M. Torrento, Carlos R. Grandini, Tiago dos S. P. de Sousa, Gerson S. de Almeida, Willian F. Zambuzzi, Diego R. N. Correa

**Affiliations:** 1 Laboratory of Anelasticity and Biomaterials, School of Sciences, Bauru Campus, São Paulo State University, Bauru 17033-360, Brazil; samuel.bonetti@unesp.br (S.P.B.); jhuliene.torrento@unesp.br (J.E.M.T.); carlos.r.grandini@unesp.br (C.R.G.); 2 Bauru Campus, São Paulo State Technological College, Bauru 17033-360, Brazil; tiago.sousa@unesp.br; 3 Department of Chemistry and Biochemistry, Institute of Biosciences, São Paulo State University, Botucatu 18618-689, Brazil; gs.almeida@unesp.br (G.S.d.A.); w.zambuzzi@unesp.br (W.F.Z.)

**Keywords:** metallic biomaterials, high entropy alloys, micro-arc oxidation, copper, biofunctionalization

## Abstract

This study evaluated the surface functionalization of a non-equiatomic TiZrNbTaMo high-entropy alloy (HEA) by micro-arc oxidation (MAO) in Cu-rich electrolytes to tailor its performance for biomedical implants. The Cu content was varied, and the resulting coatings were investigated for their morphology, phase constitution, chemical structure, wettability, and cytocompatibility. X-ray diffraction (XRD) measurements of the substrate indicated a body-centered cubic (BCC) matrix with minor HCP features, while the MAO-treated samples depicted amorphous halo with sparse reflections assignable to CaCO_3_, CaO, and CaPO_4_. Chemical spectroscopic analyses identified the presence of stable oxides (TiO_2_, ZrO_2_, Nb_2_O_5_, Ta_2_O_5_, MoO_3_) and the successful incorporation of bioactive elements (Ca, P, Mg) together with traces of Cu, mainly as Cu_2_O. MAO treatment increased surface roughness and rendered a hydrophilic behavior, which are features typically favorable to osseointegration process. In vitro cytotoxic assays with MC3T3-E1 cells (24 h) showed that Cu addition did not induce harmful effects, maintaining or improving cell viability and adhesion compared to the controls. Collectively, MAO in Cu-rich electrolyte yielded porous, bioactive, and Cu-incorporated oxide coatings on TiZrNbTaMo HEA, preserving cytocompatibility and supporting their potential for biomedical applications like orthopedic implants and bone-fixation devices.

## 1. Introduction

Bone is a dynamic, vascularized, and metabolically active tissue whose regeneration demands materials that restore function, structural integrity, and biological integration [1]. In this context, high-entropy alloys (HEAs) are a promising class of multicomponent alloys, typically composed of five or more elements in equiatomic or near-equiatomic proportions (5–35 at%), developed to overcome the limitations of existing alloys in certain engineering applications [2]. Conventionally, the developed HEAs exhibit unique characteristics, such as superior mechanical strength, corrosion and wear resistance, and thermal stability compared to conventional metallic materials, owing to the combination of configurational entropy, sluggish diffusion, lattice distortion, and cocktail effect [3]. Thus, the research and development of high-entropy materials has the potential to deliver significant advances in engineering applications.

Recently, HEAs have been considered for use as biomaterials, particularly for load-bearing implants for hard-tissue replacements. Currently, Ti-based HEAs are among the most promising alloys for medical devices, as certain compositions have proven to be biologically safe, highly corrosion- and wear-resistant in simulated body conditions, and capable of forming simple crystalline structures with high ductility [4]. These characteristics align with the typical attributes of metallic biomaterials, indicating strong potential for biomedical applications. The equiatomic Ti-Zr-Nb-Ta-Mo HEA (named as TiZrNbTaMo) has been extensively studied for biomedical implant applications [5]. For example, Hua et al. [6] conducted mechanical analyses and compared them with those of the biomedical Ti-6Al-4V alloy. Their findings indicate that the HEA performed better in biological environments due to its superior combination of properties. Furthermore, Wang and Xu [7] performed mechanical and corrosion analyses on this composition and found that the HEA demonstrated excellent corrosion resistance in PBS (Phosphate-Buffered Saline) solution, comparable to that of the Ti-6Al-4V alloy, having a remarkably superior pitting resistance than that of the SS 316L and CoCrMo alloys. However, because its surface is bioinert, surface modification techniques are crucial for enhancing interaction with adjacent bone tissues in the human body.

Among the various surface modification techniques, micro-arc oxidation (MAO) has established itself as a powerful tool for Ti-based implants [8]. The technique is focused on applying an anodic potential to the metallic surface immersed in an electrolyte, ensuring the growth of the oxide layer with the generation of plasma discharges by dielectric breakdown, resulting in a porous and thick coating, firmly adhered to the substrate, and enriched by chemical species from the electrolyte. Thus, the enrichment of the porous oxide coating with strategic chemical species has been proven to supply superior biocompatibility, osseointegration, and bioactivity [9]. In Ti-based alloys, MAO treatment has been successfully applied to produce Ca- and P-rich coatings, which are recognized for their bioactive properties that promote rapid bone recovery and growth. Furthermore, antibacterial metals (e.g., Ag, Cu, Pt, and Sr) have also been used to decorate the coatings, providing adequate bactericidal activity against diverse bacterial lineages and mitigating biofilm formation [10]. For example, Cardoso et al. [11] employed the MAO technique to incorporate Cu into the Ti-30Nb-5Mo alloy for biomedical purposes, finding that the Cu enrichment into the coatings improved the antimicrobial activity of the surface and exhibited excellent antibacterial activity against *Escherichia coli* (*E. coli*) and *Staphylococcus aureus* (*S. aureus*).

Considering that the physicochemical aspects of Ti-based HEAs are not the same as those of conventional Ti alloys, studies about the production of antibacterial coatings in HEAs by MAO treatment deserve to be explored. Accordingly, rigorous in vitro cytotoxicity evaluation is a prerequisite to any claim of antimicrobial benefit for Cu-containing MAO coatings. Complementary colorimetric assays should be employed, such as MTT, which quantifies mitochondrial dehydrogenase activity (metabolic viability), and crystal violet, which measures adherent cell biomass (attachment/proliferation) [12,13]. Used in 24–72 h exposure and dose–response formats with appropriate controls, these orthogonal endpoints increase sensitivity to sublethal mitochondrial dysfunction and anti-adhesive effects, reduce false negatives, and enable definition of biocompatible materials in accordance with ISO 10993-5/-12 principles and justify further progression to in vivo evaluation as we have widely discussed [14,15,16].

Here, we focused on functionalizing the surface of the TiZrNbTaMo HEA by applying MAO treatment in a Cu-rich electrolyte to probe the influence of Cu on the resulting oxide coating and its suitability for biomedical implants. Following MAO, specimens were systematically characterized for surface topography, chemical composition, phase constitution, roughness, wettability, Vickers microhardness, and in vitro cytotoxicity, enabling a comprehensive assessment of Cu-mediated effects on coating properties and biocompatibility.

## 2. Materials and Methods

### 2.1. Sample Processing

An ingot (30 g) was produced from commercially pure metals (Ti, Zr, Nb, Ta, and Mo; purity > 99%) that had been previously separated into equiatomic proportions. The metals were ultrasonically cleaned in an acetone bath for 10 min (0.6 ks). Afterward, the metals were melted in an arc-melting furnace under an inert argon atmosphere, using a non-consumable tungsten electrode and a water-cooled copper crucible. The ingot was remelted 7 times to ensure complete mixing of the alloying elements. Then, the ingots underwent a homogenization heat treatment at 10^−6^ Torr, with a heating rate of approximately 10 K·min^−1^, a temperature plateau of 1273 K for 12 h (43.2 ks), followed by slow cooling to room temperature.

### 2.2. Surface Treatment

The ingot was sectioned into approximately 2 mm-thick lamellar samples using a cutting machine (ISOMET 100, USA). Subsequently, the samples were cold-mounted in acrylic resin and ground on waterproof SiC paper (mesh sizes up to 1200). Before surface treatment, the samples were removed from the resin and cleaned in an ultrasonic acetone bath for 10 min, followed by another cleaning cycle in distilled water. The MAO treatment was conducted at 300 V, with a limited current of 2.5 A, for 0.6 ks. A digital multimeter (Keysight, model N5751A, Santa Rosa, CA, USA) and a computer were used to record the current through the sample during the process. The electrolyte was composed of distinct proportions of calcium acetate (CaA; Ca(C_2_H_3_O_2_)_2_), β-calcium glycerophosphate (β-GP; C_3_H_7_CaO_6_P), magnesium acetate (MgA; Mg(C_2_H_3_O_2_)_2_), and copper acetate (CuA; Cu(C_2_H_3_O_2_)_2_). The electrical parameters and electrolyte composition were set up from our earlier studies in the literature [10]. The electrolyte was previously magnetically stirred for 1 h (3.6 ks) to ensure a complete mixing of the reagents. The samples and their corresponding electrolyte compositions are presented in Table 1.

### 2.3. Sample Characterization

For microstructural analysis, the samples were polished in an alumina suspension (0.25 μm) and immersed in an aqueous solution containing 10% nitric acid (HNO_3_) and 5% hydrofluoric acid (HF) for approximately 5 s to reveal the microstructure. Microstructural and topographical analyses were performed using an optical microscope (Olympus Inc., model BX51M, Center Valley, PA, USA). A scanning electron microscope (SEM; Carl Zeiss, model LS15 EVO, White Plains, NY, USA) was also used for imaging in secondary (SE) and backscattered (BSE) electron beam modes, with a voltage range of 15–20 kV. Structural analysis was carried out using X-ray diffraction (XRD) measurements with a Rigaku diffractometer, model MiniFlex 600, operating at 15 mA and 40 kV, in fixed time mode, with a Bragg–Brentano (θ-2θ) configuration, a step of 0.04°, a collection time of 1.6 s, and monochromatic Cu-Kα radiation (λ = 1.540456 Å). The HighScore Plus^®^ software version 3.0 analyzed the diffracted peaks using crystallographic data sheets from the Crystallography Open Database (COD).

Semi-quantitative chemical microanalysis was conducted using energy-dispersive X-ray spectroscopy (EDS; Bruker, Billerica, MA, USA) in a detector coupled to the SEM equipment, operating at 15 kV, Fourier-transform infrared spectroscopy (FTIR; JASCO Corp, model FT/IR-410, Easton, MD, USA) operating in absorbance mode at room temperature, with 120 scans and a resolution of 2 cm^−1^, and Raman spectroscopy (Metrohm Raman spectrometer, model i-Raman Plus 532H, Laramie, WY, USA) operating with wavelength of 532 nm and laser power of 100%. Further chemical details were acquired by X-ray photoelectron spectroscopy (XPS; Thermo Scientific, model Thermo K-alpha, Waltham, MA, USA) with an Al-Kα radiation source (1.486 eV), spot size of 400 μm, energy step of 200 eV, for long scan (survey), and 50 eV, for short scan spectra (high-resolution), with resolutions of 1 eV and 0.01 eV, respectively. The energy correction was applied using the C1s peak. The results were analyzed using CasaXPS^®^ version 2.3.24 software.

Roughness values (Ra—average roughness and Rq—root mean square average roughness) were obtained by laser confocal microscopy (DCM3D, Leica, Teaneck, NJ, USA), with a resolution of 0.160 µm and a measurement range of 524 µm. The surface wettability was evaluated by measuring contact angles using a Ramé-Hart goniometer (Ramé-Hart Instrument Inc., model 100-00, Succasunna, NJ, USA), with distilled water and diiodomethane droplets (20 µL) at room temperature. Average values were obtained from three randomly selected regions along the surface. Vickers microhardness values were obtained using a Shimadzu HMV-G instrument (Marlborough, MA, USA), with a load of 2.942 N and a dwell time of 15 s. Average values were calculated from 10 random measurements along the surface.

### 2.4. Biological Evaluation

Cell viability and adhesion were analyzed using the MTT (3-(4,5-dimethylthiazol-2-yl)-2,5-diphenyltetrazolium bromide) and crystal violet (CV) colorimetric assays, where MC3T3-E1 mouse pre-osteoblastic cells (subclone 4; ATCC CRL-2593) were cultured in α-MEM supplemented with 10% fetal bovine serum (FBS) at 37 °C and 5% CO_2_. Sub-confluent cultures were trypsinized and used in all experiments.

For the cell viability assay, α-MEM (without FBS) was conditioned with the materials at 0.2 g/mL for 24 h at 37 °C. After conditioning, FBS was added to a final concentration of 10%, and the conditioned medium was used to treat cells. Cells were seeded at 5 × 10^4^ cells/mL in 96-well plates, and after 24 h, the culture medium was replaced with the conditioned medium, and cells were incubated for an additional 24 h (n = 6). Internal control was assayed by keeping the cells exposed to conventional culture medium (control). After 24 h of treatment with conditioned medium, the medium was replaced with fresh conventional medium containing 1 mg/mL MTT reagent and incubated for 3 h. The formed formazan crystals were dissolved in 0.1 mL of dimethyl sulfoxide (DMSO) in each well, and cell viability was estimated by measuring absorbance at 570 nm using a microplate reader (SYNERGY-HTX multi-mode reader, Biotek, Winooski, VT, USA).

For the cell adhesion assay, pre-osteoblast cells were seeded at 5 × 10^4^ cells/well in 96-well plates using conditioned medium supplemented. After 24 h, adherent cells were rinsed in warm phosphate-buffered saline (PBS) and then fixed in a 3:1 (*v*/*v*) mixture of absolute ethanol and glacial acetic acid for 10 min at room temperature. The adherent cells were stained with 0.1% crystal violet (*w*/*v*) for 10 min at room temperature. Excess dye was removed by decantation, and the sample whas washed twice with distilled water. The dye was extracted with 10% acetic acid (*v*/*v*), and the optical density was measured at 540 nm using a microplate reader (Biotek Co., Winooski, VT, USA). Data from each experiment were analyzed with six observations per group (n = 6).

Statistical analyses were performed using GraphPad Prism 7 software (GraphPad Software, San Diego, CA, USA). Data were analyzed using two-way analysis of variance (ANOVA) followed by Tukey’s post hoc test for multiple comparisons. When data did not meet the assumptions of normality and homoscedasticity, non-parametric tests were employed. In all cases, a *p*-value < 0.05 was considered statistically significant.

## 3. Results

**Figure 1:**
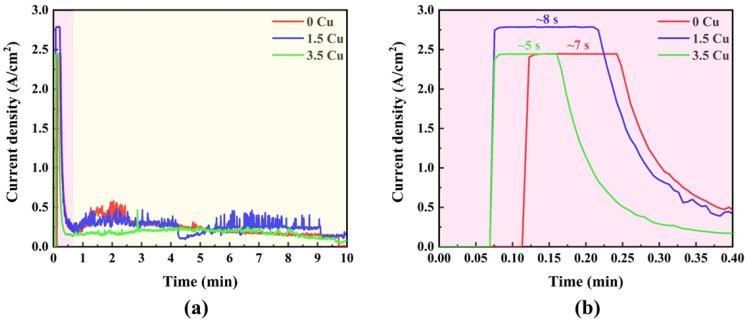
Current density as a function of time: overall (**a**) and zoomed view (**b**).

The current density vs. time plot of the MAO-treated samples is shown in Figure 1. In the first seconds (Figure 1a), there was a sharp decay in the current density in all samples, beginning around 2.0–3.0 A/cm^2^ and achieving values below 0.5 A/cm^2^. Subsequently, the current densities remain at a plateau, with minimal temporal fluctuations. The zoomed view (Figure 1b) indicates that the initial decay is not uniform for all samples, exhibiting a nonlinear dependence on the Cu concentration in the electrolyte. As previously reported [12], the initial decay is due to rapid oxide growth induced by the anodic voltage, which acts as an insulating barrier (dielectric) and prevents charge transfer from the electrolyte to the substrate. As charges accumulate in the coatings, dielectric breakdown eventually occurs, resulting in plasma discharges (micro-arcs) and gas bubble formation. Therefore, the noise in the plateau stage can be linked with the corresponding micro-arcs along the coating. This finding is consistent with some similar studies in the literature [11,13], which found that increasing the Cu concentration in the electrode led to faster dielectric breakdown. This is consistent with the present result, as the first dielectric drop occurred on the electrode with the highest Cu concentration.

**Figure 2:**
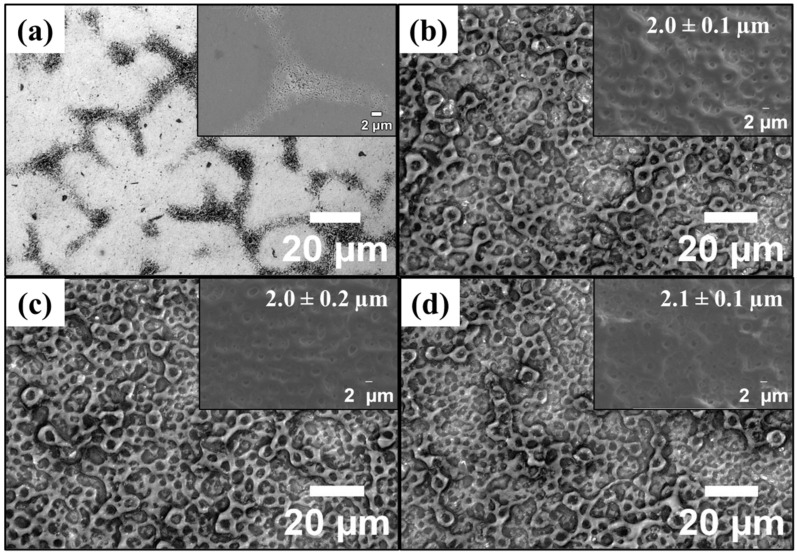
Microstructure of the substrate (**a**), and topographical view of the MAO-treated samples: 0 Cu (**b**), 1.5 Cu (**c**), and 3.5 Cu (**d**). SEM imaging and average pore size as an inset.

The microstructure of the substrate and the corresponding topography of the MAO-treated samples are shown in Figure 2. Overall, the substrate depicted some bright granular microstructure related to a TaMoNb-rich BCC phase and some dark precipitates in the boundaries, related to TiZr-rich HCP phase, agreeing well with our previous studies [14]. All MAO-treated samples exhibited a typical porous coating, with no evidence of microscale cracking. The average pore size remained approximately 2.0 µm across all Cu concentrations, suggesting that the plasma energy and distribution were unchanged. As in the study by Yao et al. [15], no obvious differences in low-magnification morphology were observed between the Cu-doped and Cu-free coatings, indicating that Cu incorporation had no significant effect on the coating topography.

**Figure 3:**
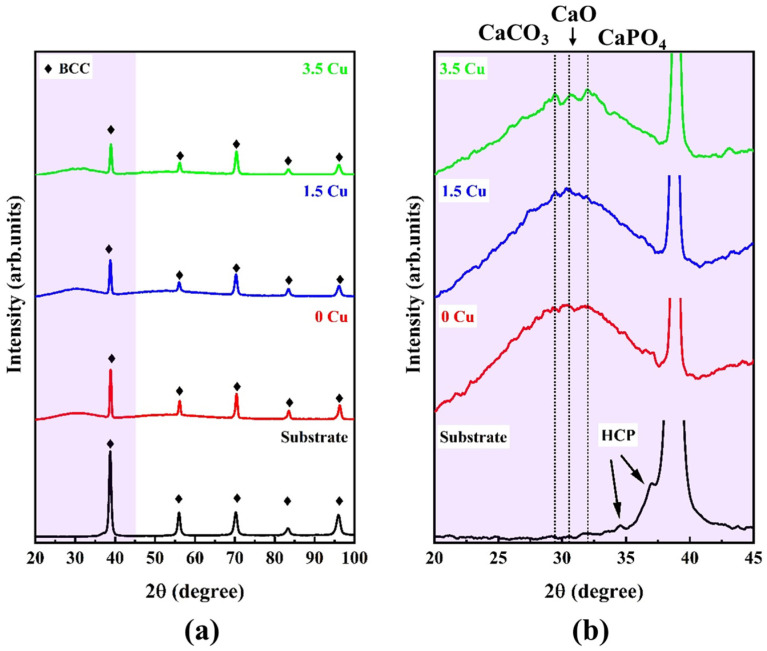
XRD profile of the studied samples in an overall (**a**) and zoomed view (**b**).

The phase proportions in the substrate and coatings are shown in Figure 3. The substrate exhibited sharp peaks from the BCC phase (CIF #1523154), with some sparse peaks from the HCP phase (CIF #9008523) visible in the zoomed view, which agreed well with the phase proportions identified in Figure 2. The MAO-treated samples depicted a pronounceable halo from amorphous phase below 40°, which was probably formed during the dielectric breakdown and plasma discharges of the surface treatment. However, in the zoomed view, it was possible to identify some sparse peaks from the CaCO_3_ (CIF #1010962), CaO (CIF #1011095), and CaPO_4_ (CIF #1517238) phases, which could have resulted from the Ca- and P-enrichment in the outer layer. Although the overall XRD profiles remained unchanged with increasing Cu concentration, the intensities of the BCC phase peaks decreased, indicating the presence of a thick surface layer. Similar results were detected by Sousa et al. [10], who found the same amorphous halo when applying MAO treatment to the TiZrNbTaMo HEA in a Cu-free electrolyte.

**Figure 4:**
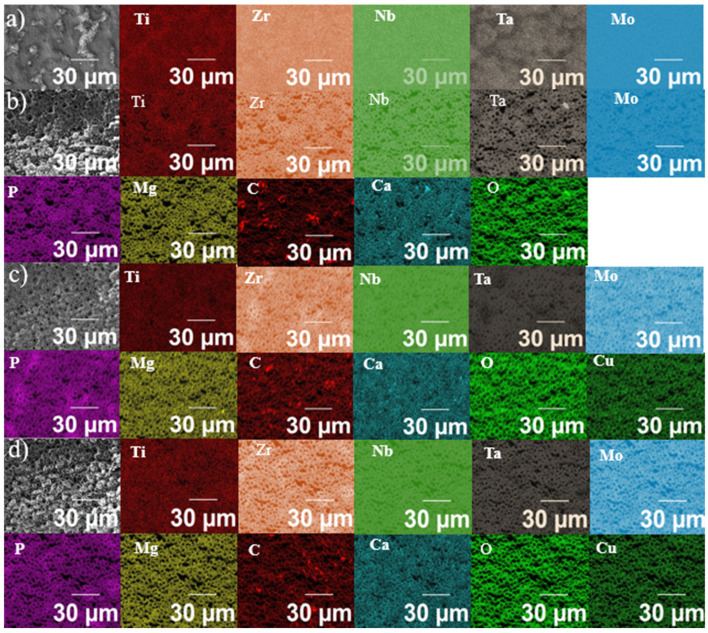
Elemental EDS map of the samples: substrate (**a**), 0 Cu (**b**), 1.5 Cu (**c**), and 3.5 Cu (**d**).

**Figure 5:**
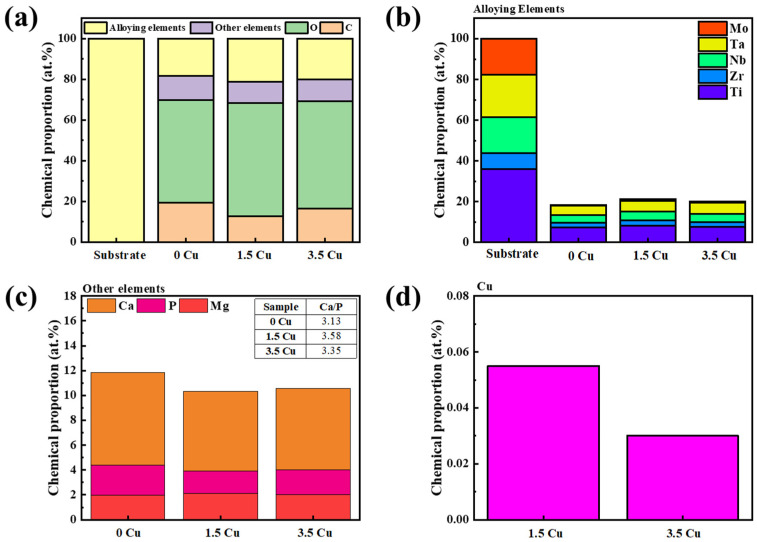
Semi-quantitative EDS analysis of the samples: overall elements (**a**), alloying elements (**b**), bioactive elements (**c**), and Cu (**d**).

The elemental EDS analysis of the samples is shown in Figure 4. The substrate exhibited a chemical segregation within the existing phases, with Ta enriched inside the grains and Ti enriched at the grain boundaries, consistent with our previous studies [14]. Moreover, the MAO-treated samples exhibited a uniform distribution of the selected elements across the coatings, with no clear differences in Cu content. Chemical details are presented in the semi-quantitative EDS analysis, performed in the selected-area mode and shown in Figure 5. The overall chemical composition (Figure 5a) indicates that the MAO-treated samples contain considerable amounts of O and C, likely as oxides and carbonates, respectively. A look at the alloying elements (Figure 5b) indicated that the proportion decreased significantly with the MAO treatment, with most of the Ti remaining, along with a secondary amount of Ta, Nb, and Zr, and Mo as a trace. Therefore, although the bulk had an equiatomic chemical composition, the MAO coating exhibited distinct elemental abundances, mainly due to differences in the oxidation of the alloying elements. Specifically for Ti, which are more reactive with oxygen and tend to oxidize preferentially, forming stable oxides. Regarding the number of bioactive species (Figure 5c), although Mg concentration remained nearly constant, Ca and P exhibited fluctuations under each condition, suggesting that Cu influenced chemical incorporation of species from the electrolyte. The prevalence of Ca compared to Mg and P is directly related to its abundance in the electrolyte, resulting in a Ca/P ratio higher than that expected for the hydroxyapatite (1.67). Lastly, in Figure 5d, the amount of Cu incorporated into the MAO coating can be compared. Interestingly, its chemical proportion decreased with increasing electrolyte concentration, supporting the assumption that Cu addition can influence electrical conductivity and favor dielectric breakdown and plasma discharges during MAO treatment. Moreover, the Cu concentration remained at a trace level in the MAO coatings (<0.1 at%), suggesting the potential to affect microorganisms without interfering with cells or tissues. As reported by Zong et al. [16], excessive Cu doping in nanotubes can be toxic to osteoblasts. However, cytotoxicity was negligible when the Cu content was relatively low, thereby enabling integration between the favorable antibacterial activity of Cu and promising biological properties.

**Figure 6:**
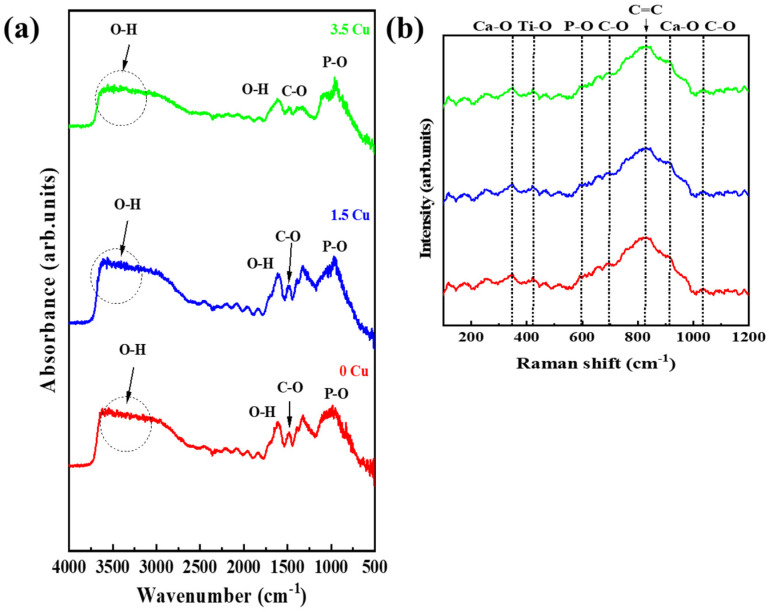
Vibrational chemical analysis: FTIR spectra (**a**) and Raman spectra (**b**).

FTIR and Raman spectroscopy enabled the identification of molecular radical groups in the coatings. Overall, the FTIR spectra (Figure 6a) of all MAO-treated samples were similar, with minor differences observed at low wavenumbers. The broad band detected around 3400 cm^−1^ is attributed to the O–H stretching vibration, indicating the presence of hydroxyl groups and/or adsorbed water molecules within the porous surface. While the weak band near 1650 cm^−1^ corresponds to the H–O–H bending vibration of adsorbed water. The absorption peak detected around 1450 cm^−1^ is assigned to the asymmetric stretching mode of carbonate ions (CO_3_^2−^), while the intense band centered between 1050 and 1000 cm^−1^ corresponds to the asymmetric stretching vibration of phosphate groups (PO_4_^3−^). These bands confirmed the presence of carbonate and phosphate compounds in the surface, as indicated in the XRD results. These bands were also identified in studies involving MAO treatment in Ti alloys that incorporated calcium and phosphorus similarly [17,18]. In the Raman spectrum (Figure 6b), P-O bands were also identified, agreeing with those reported by Stammeier et al. [19]. Additionally, Ti-O, Ca-O, C=C, and C-O bands were also identified and corroborated the literature [17,20], supporting the results obtained by FTIR.

**Figure 7:**
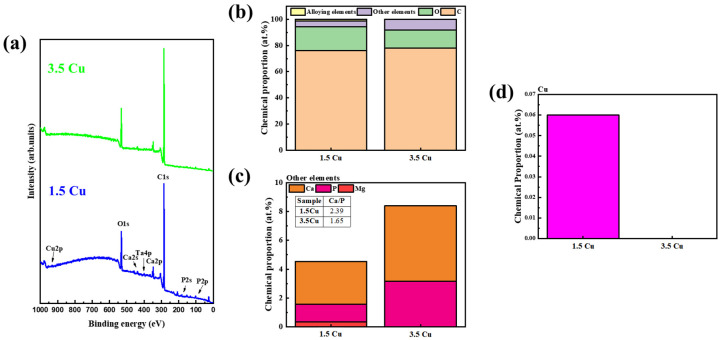
XPS analysis of the Cu-rich MAO-treated samples: survey spectra (**a**), semi-quantitative chemical proportion of all elements (**b**), bioactive elements (**c**), and Cu (**d**).

XPS analysis was performed on Cu-rich MAO-treated samples to provide further details on the chemical composition of the outer layer. The survey spectra (Figure 7a) and the corresponding semi-quantitative analysis (Figure 7b) indicated the abundance of C and O in the outer layer, likely due to the presence of oxides, organic, and inorganic compounds formed during MAO treatment and atmospheric exposure. Ca and P elements (Figure 7c) were gradually enriched with the addition of Cu to the electrolyte, but the Ca/P ratio differed from that observed in the EDS results. Given that EDS analysis has a greater penetration depth, it can be inferred that Ca and P are non-uniformly distributed in the MAO coating. Another relevant factor is that in the 3.5 Cu sample, neither Mg nor Cu (Figure 7d) was detected by XPS, indicating that these elements are in deeper regions of the coating. High-resolution XPS spectra (Supplementary Material) revealed the presence of inorganic compounds (phosphate, CaCO_3_, and MgCO_3_), similar to those previously identified by our group [21,22]. The Cu was found as Cu_2_O, indicating a possible forming chemical reaction into the electrolyte, such as $2Cu{\left(CH_{3}COO\right)}_{2}+H_{2}O\to {Cu}_{2}O+4CH_{3}COOH$. Further evaluation of the cross-sectional view of the MAO-treated samples can provide valuable information on the elemental distribution along the coating. Furthermore, the C1s spectra were deconvoluted into three main components (C-C, C-O, and O=C), confirming the presence of carbonates, phosphates, and oxides in the coatings. Even at low concentrations, it was also possible to notice the presence of some alloying elements from the bulk in their more stable oxide forms (TiO_2_, ZrO_2_, Nb_2_O_5_, Ta_2_O_5,_ and MoO_3_), identical to those found by Sousa et al. [10].

**Figure 8:**
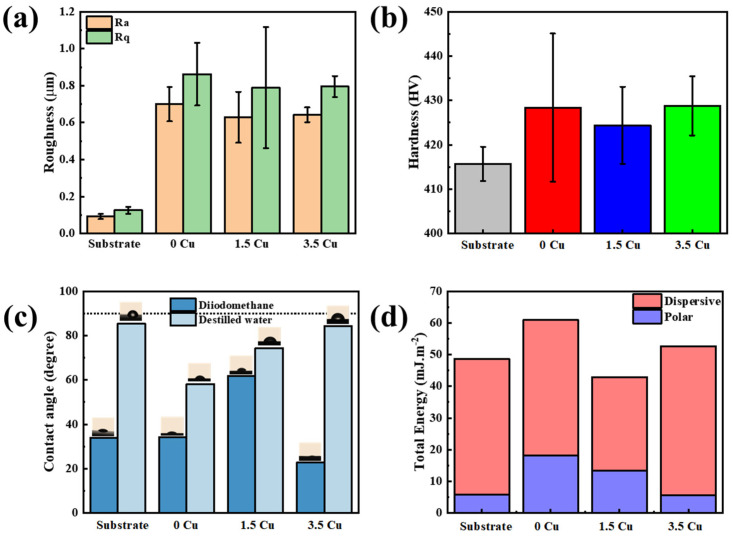
Physicochemical characteristics of the surfaces: roughness (**a**), Vickers microhardness (**b**), contact angle (**c**), and surface energy (**d**). (Dotted line in (**c**) refers to the 90 degree value).

The MAO-treated samples (Figure 8a) exhibited rougher surfaces than the substrate, due to micro-arc discharges and pore formation. The addition of Cu slightly reduced roughness, though further studies are needed to confirm this effect. Roughness values remained in the micrometer range, which is beneficial for fixation of bone tissues, as demonstrated by Wennerberg et al. [23] in dental implants. The authors reported that implants with an average roughness of 0.9–1.3 μm showed better fixation than those with 0.4 μm. The hardness values of the MAO-treated samples (Figure 8b) were slightly higher than those of the substrate, due to the presence of the oxide layer on the surface. The incremental amount of Cu in the coatings did not significantly affect the Vickers microhardness. Regarding the contact angle measurements (Figure 8c), all samples exhibited hydrophilic behavior (<90°) with a nonlinear dependence on Cu content, supporting the idea that the presence of metal in the electrolyte can influence the oxidation process and the growth mechanisms of the coating. This hydrophilic character is relevant, as it is typically associated with surface bioactivity and cell adhesion. Implants with hydrophilic surfaces are known to promote enhanced responses from osteogenic cells, facilitating early osseointegration and potentially increasing the overall success rate of implantation [24,25]. However, surfaces with lower hydrophilicity may also confer advantages for implants, as they tend to mitigate bacterial adhesion [25,26]. The corresponding surface energy values (Figure 8d) primarily reflected a dispersive component, consistent with XPS analyses that reported a considerable presence of organic compounds in the outer layer. The polar component characterizes strong bonds between molecules (hydrogen, ionic, covalent, dipole–dipole bonds), while the dispersed component characterizes weak bonds of dispersion forces (van der Waals interactions). As reported by Hamraoui [27], high surface energy can promote a cascade of biological responses, including protein adsorption, cell behavior, and bacterial attachment.

**Figure 9:**
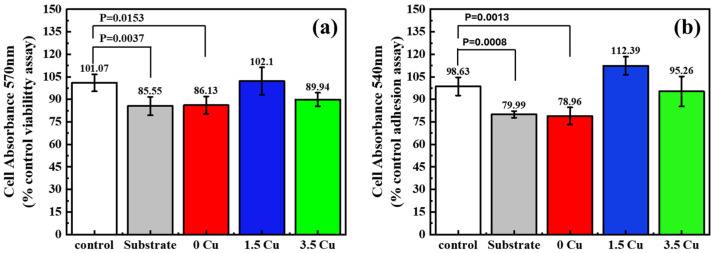
In vitro cytotoxic results of the samples: MTT (**a**) and CV (**b**) assays.

The preliminary cytotoxicity analysis (Figure 9) indicates that adding Cu to the electrolyte did not affect initial cell viability or adhesion. Overall, values were comparable to or higher than the control, indicating that the tested Cu levels were non-cytotoxic. Further studies may demonstrate the antimicrobial action of Cu, as previous research has shown that even low concentrations can exhibit antibacterial properties. As an example, Shimabukuro et al. [28] showed that minor amounts of Cu incorporated into MAO coatings grown on commercially pure titanium possessed no harmful effects on the proliferation and calcification of osteoblast-like cells. Moreover, anaerobic bacterial growth was inhibited in these samples. The study also revealed that antibacterial activity against *S. aureus* was prolonged and enhanced upon immersion in physiological saline solutions.

## 4. Conclusions

In this study, the non-equiatomic TiZrNbTaMo HEA was subjected to MAO treatment in Cu-rich electrolytes to functionalize the surface for biomedical applications. From the results obtained, it is possible to summarize the main findings as follows:

- The Cu concentration modulated MAO stages, altering the timing of dielectric breakdown and plasma discharges.
- Untreated material exhibited a granular BCC matrix with small HCP precipitates at grain boundaries, whereas MAO-treated surfaces displayed the characteristic porous topography, with microscale round pores.
- XRD patterns of the MAO-treated samples exhibited a pronounced amorphous halo with sparse reflections assigned to CaCO_3_, CaO, and CaPO_4_.
- Chemical analyses confirmed preferential Ti oxidation despite the other alloying elements, the incorporation of bioactive species (Ca, P, Mg), and decoration of Cu ions into the coatings.
- All MAO-treated samples depicted a rough surface, with hydrophilic behavior and a majority of dispersive surface energy component, which are promising to promote cell adhesion and proliferation.
- Preliminary in vitro assays collected after 24 h showed that Cu addition did not induce cytotoxicity and maintained or improved cell viability and adhesion relative to controls, suggesting that Cu-enriched coatings preserve biocompatibility while potentially conferring antibacterial functionality.
- Collectively, the study shows that MAO treatment of HEAs in Cu-rich electrolyte can be a promising pathway to surface engineer biomedical implants, especially those for long-term usage. Further evaluation of the mechanical, electrochemical, and antibacterial properties can provide a basis for screening potential medical applications.

## Figures and Tables

**Table 1:** Nomenclature of the samples and the corresponding composition of the electrolyte.

Sample	Electrolyte			
	CaA (mol·L^−1^)	β-GP (mol·L^−1^)	MgA (mol·L^−1^)	CuA (mol·L^−1^)
0 Cu	0.35	0.02	0.10	0.00
1.5 Cu				1.50
3.5 Cu				3.50

## Data Availability

The original contributions presented in the study are included in the article. Further inquiries can be directed to the corresponding author.

## Supplementary Materials

The following supporting information can be downloaded at https://www.mdpi.com/article/10.3390/ma19010174/s1, Figure S1: HR spectrum of studied elements.; Table S1: Quantitative results of the HR XPS analysis.

## Abbreviations

The following abbreviations are used in this manuscript:

HEA	High-entropy alloy
MAO	Micro-arc oxidation
BCC	Body-centered cubic
HCP	Hexagonal close-packed
FTIR	Fourier transform infrared
XPS	X-ray photoelectron spectroscopy
EDS	X-ray energy-dispersive spectroscopy
XRD	X-ray diffraction
SEM	Scanning electron microscopy
