# Supplementary material for: Surface Engineering of Non-Equiatomic TiZrNbTaMo HEA by MAO Treatment in a Cu-Rich Electrolyte for Biomedical Applications
Source: Materials (Basel). 2026 Jan 3;19(1):174. doi: 10.3390/ma19010174 (PMC12786585; doi:10.3390/ma19010174)
Supplement: Supplementary file 1 [file materials-19-00174-s001.zip › materials-4004192-supplementary.pdf]

Supplementary material

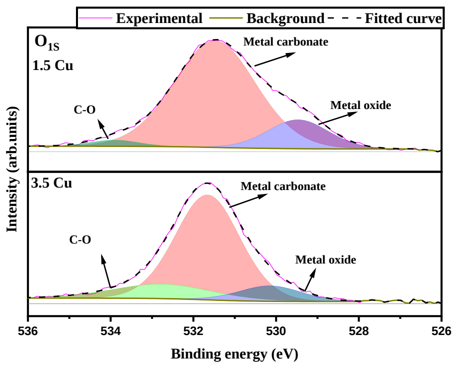

O1s

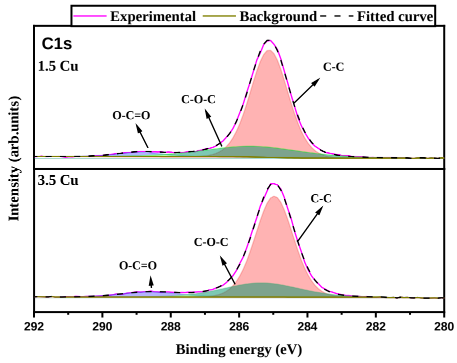

C1s

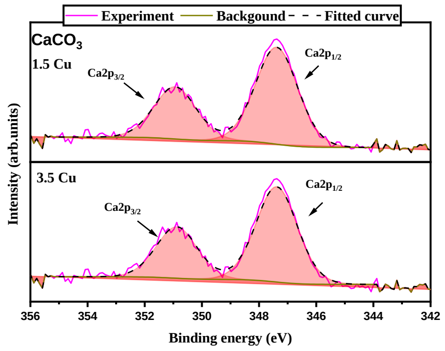

Ca2p

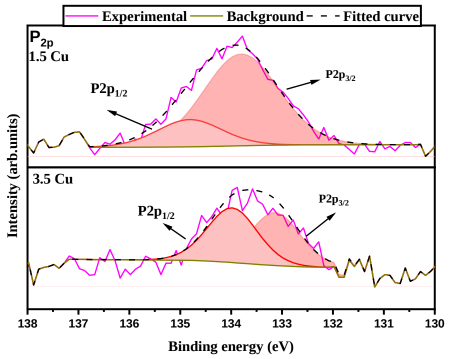

P2p

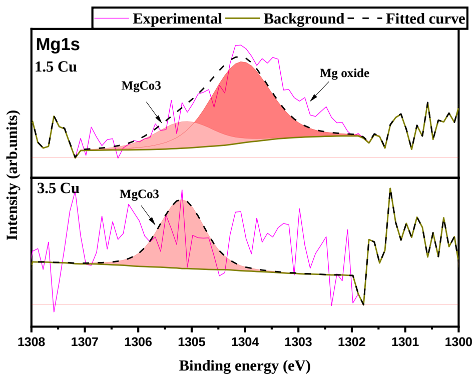

Mg1s

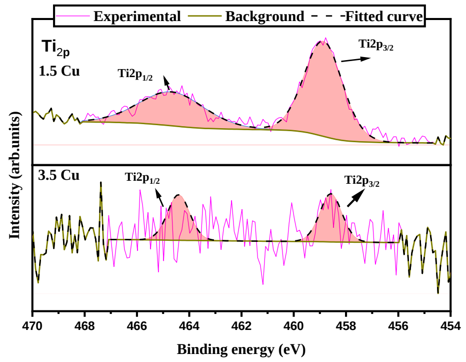

Ti2p

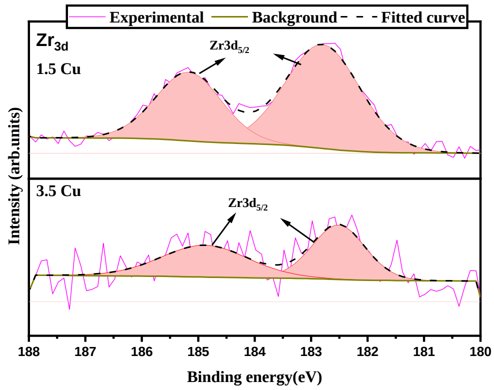

Zr3d

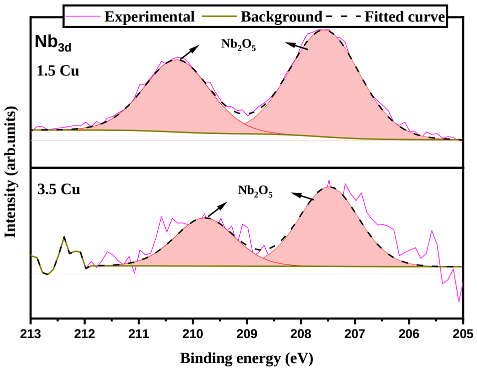

Nb3d

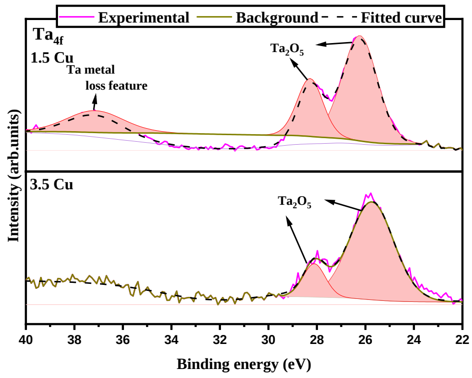

Ta4f

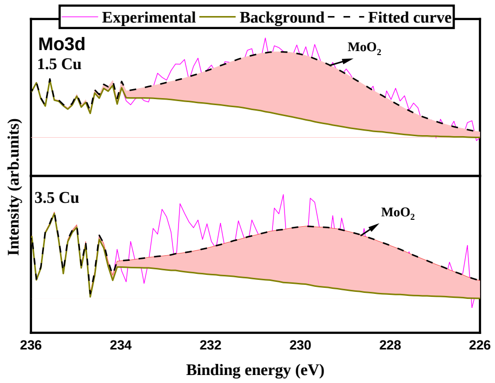

Mo3d

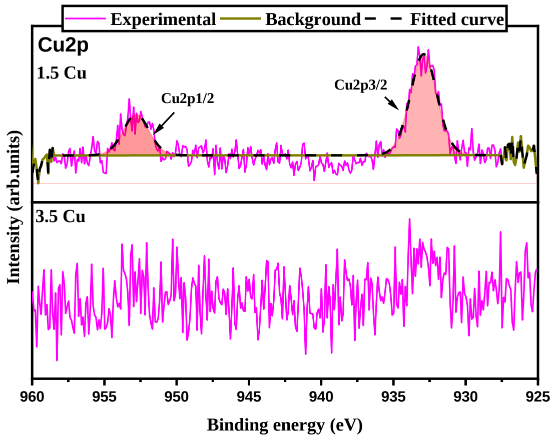

Cu2p

Fig. S1: HR spectrum of studied elements.

Table S1: Quantitative results of the HR XPS analysis.

Sample	Spectrum	Residual	Chemical designation	Proportion (%)	Peak position (eV)
**1.5 Cu**	O1s	1.13	Metal carbonate	80.81	531.68
			C-O	2.82	534.18
			Metal oxide	16.73	530.08
	Ti2p	0.90	TiO_2	100.00	458.88 ($p_{3/2}$)<br>464.78 ($p_{1/2}$)
	Zr3d	0.95	ZrO_2	100.00	182.70
	Nb3d	1.15	Nb_2O_5	100.00	207.18
	Ta4f	1.14	Ta_2O_5	80.80	26.28
			Ta metal loss feature	19.20	37.18
	Mo3d	1.07	MoO_3	100.00	229.68
	C1s	1.07	C-C	77.00	288.98
			C-O	19.52	286.18
			O=C-O	3.49	285.08
	Ca2p	0.91	$CaCO_3$	100.00	347.28 ($p_{3/2}$)<br>350.88 ($p_{1/2}$)
	P2p	1.11	PO_4^{3-}	100.00	133.78 ($p_{3/2}$)<br>134.88 ($p_{1/2}$)
	Mg1s	0.84	$MgCO_3$	24.78	1305.28
			MgO	75.22	1304.08
	Cu2p	1.09	Cu_2O	100.00	932.88($p_{3/2}$)<br>952.68($p_{1/2}$)
**3.5 Cu**	O1s	0.87	Metalcarbonate	74.02	531.68
			C-O	10.41	533.28
			Metal oxide	15.57	530.08
	Ti2p	1.12	TiO_2	100.00	458.63 ($p_{3/2}$)<br>464.34 ($p_{1/2}$)
	Zr3d	1.15	ZrO_2	100.00	182.48
	Nb3d	1.04	Nb_2O_5	100.00	207.48
	Ta4f	1.07	Ta_2O_5	100.00	25.78
	Mo3d	1.05	MoO_3	100.00	229.98
	C1s	1.11	C-C	73.92	288.88
			C-O	20.42	285.68
			O=C-O	5.69	288.58
	Ca2p	1.02	$CaCO_3$	100.00	347.30 ($p_{3/2}$)<br>350.88 ($p_{1/2}$)
	P2p	0.94	PO_4^{3-}	100.00	133.31 ($p_{3/2}$)<br>134.02 ($p_{1/2}$)
	Mg1s	0.88	$MgCO_3$	100.00	1305.28
